# Effect of dentition status on perceived mobility limitation among older Brazilian adults: a cohort study

**DOI:** 10.1590/1807-3107bor-2025.vol39.086

**Published:** 2025-09-08

**Authors:** Fabiola Bof de ANDRADE, Renata Lara FREITAS, Yeda Aparecida de Oliveira DUARTE, Jair Lício Ferreira SANTOS

**Affiliations:** (a) Fundação Oswaldo Cruz, Rene Rachou Institute, Belo Horizonte, MG, Brazil.; (b) Universidade de São Paulo – USP, Nursing School, Sao Paulo, SP, Brazil.; (c) Universidade de São Paulo – USP, School of Medicine of Ribeirão Preto, Ribeirão Preto, SP, Brazil.

**Keywords:** Oral Health, Tooth Loss, Mobility Limitation, Adults, Persons with Disability

## Abstract

This study aimed to evaluate the longitudinal effect of dentition status on the perceived mobility limitation of community-dwelling Brazilian older adults. This cohort study used data from individuals who participated in the second (2006), third (2010), and fourth (2015) waves of the Health Well-being and Aging Study, conducted in the urban region of the city of São Paulo, Brazil, with adults aged 60 years and older. Mobility limitation was assessed in all waves according to reports of difficulty in performing seven activities, with higher scores representing a higher number of limitations. The independent variables of interest were number of teeth, use of dental prostheses, impact of oral health on functionality, and presence of periodontal pockets. Oral health measures were assessed by dentists, in all waves, during a clinical oral examination. The generalized linear mixed model with a Poisson distribution was used to assess longitudinal associations. All the variables were treated as time-varying in the analysis. Older adults with 20 or more teeth had a lower risk of mobility limitation than edentulous individuals, while the impact of oral health on functionality was associated with an increased risk. Similar findings were observed among dentate individuals. Periodontal disease was not associated with the outcome in dentate individuals. The associations were constant over time. The number of teeth and the impact of oral health on functionality are risk factors for mobility limitation, underscoring the importance of maintaining functional dentition for healthy aging.

## Introduction

Mobility refers to all forms of movement, whether powered by the body or a vehicle, and extends beyond mere physical movement.^1,2^ The ability to be mobile is one of the five domains of functional ability that is essential for healthy aging.^3^ The significance of mobility in the lives of older adults is profound, being closely related to independence, quality of life, and the ability to engage in social and community activities.^1,3^ Conversely, mobility limitations are associated with increased dependency^3^ and social isolation.^4^ Mobility limitation in older adults is increasingly recognized as an early indicator of difficulties in activities of daily living.^5^ Thus, understanding the factors contributing to mobility limitation is crucial in geriatric healthcare and public health planning.

The World Health Organization defines oral health as the state of the mouth, teeth, and orofacial structures that enable individuals to perform essential functions, such as eating, breathing, and speaking. It also encompasses psychosocial dimensions, such as self-confidence, well-being, and the ability to socialize and work without pain, discomfort, and embarrassment.^6^ Given this definition, oral health plays a critical role in maintaining the functionality of essential activities directly related to mobility limitation. While the potential link between oral health and disability is not fully understood, there is evidence suggesting that poor oral health can lead to malnutrition^7^ and systemic inflammation,^8,9^ both of which are known risk factors for disability.^10,11^

Previous research has established associations between poor oral health and various health conditions, including cardiovascular diseases, respiratory infections, diabetes,^8^ and disability.^12^ However, the specific impact of dentition status on mobility limitations has been less extensively explored, particularly in contexts such as those of developing countries. Evidence shows that tooth loss^12,13^ and periodontal disease^12^ have been associated with the incidence of mobility limitation. Nevertheless, the findings concerning the number of teeth related to the outcome were not consistent across studies. These studies evaluated dentition status at baseline and encompassed different populations of older adults aged 70 years or older, including individuals from health services in Denmark,^13^ white British men from various towns across the UK, and white and African American participants from Memphis and Pittsburgh in the United States.^12^ Although perceived mobility was the outcome in the three samples, measured through self-reported ability or difficulty with mobility task performance,^2^ the differences in measurement methods across the studies hinder direct comparisons. Thus, longitudinal studies that include a wider range of age groups, a comprehensive number of oral health measures, and covariates are important for better understanding this association. Additionally, the role of biomarkers in this association is not known.

This study aimed to evaluate the longitudinal effect of dentition status on the perceived mobility limitation among community-dwelling Brazilian older adults.

## Methods

### Study design, population, and data source

This cohort study used data from individuals who participated in the second (2006), third (2010), and fourth (2015) waves of the Health Well-being and Aging Study (*Saúde Bem-Estar e Envelhecimento* (in Portuguese) - SABE).

The SABE study started in the year 2000 under the guidance of the Pan American Health Organization (PAHO) and was a pioneering research initiative designed to shed light on the health conditions, overall well-being, and aging trajectories of older adults in Latin America and the Caribbean. In Brazil, the study took place in the city of São Paulo and had a representative sample of community-dwelling older adults aged 60 and older.

### Sampling

The study waves took place at roughly five-year intervals. For each successive wave of the study, efforts were made to re-interview all previously identified participants who could be reached, alongside the inclusion of a new cohort of individuals aged 60 to 64 years. This approach was adopted to maintain the representativeness of this specific age group within the overall study population. The participants across all samples were recruited through a two-stage stratified random sampling method. Initially, the selection process involved census tracts, followed by the identification of households within these tracts.

The initial sample in 2000 was created using this two-stage stratified random sampling approach, based on geographic regions outlined by the 1995 National Household Survey. To account for higher mortality rates and lower sampling probabilities among those individuals aged 75 years and older, an additional sample from this age group was included. In 2000, 2,143 older adults were interviewed. By 2006, 1,115 participants had been re-interviewed, and 298 new individuals aged 60 to 64 years had been added through probabilistic randomized sampling, resulting in a sample of 1,413 individuals. In 2010, 915 older participants were re-interviewed, and 355 new participants aged 60 to 64 years were included, totaling 1,333 interviews. In 2015, the sample included 838 re-interviewed participants and 386 new individuals aged 60 to 64 years, yielding 1,224 interviews. All interviews were conducted in participants’ homes. Detailed information about the study design and sampling procedures has been previously published.^14^

We used data from the second wave onwards, considering that clinical oral health examinations were not available in the first wave. Only participants with complete information for all variables of interest were included in the analyses. Figure illustrates the study sample included in each wave of the present study.

**Figure:**
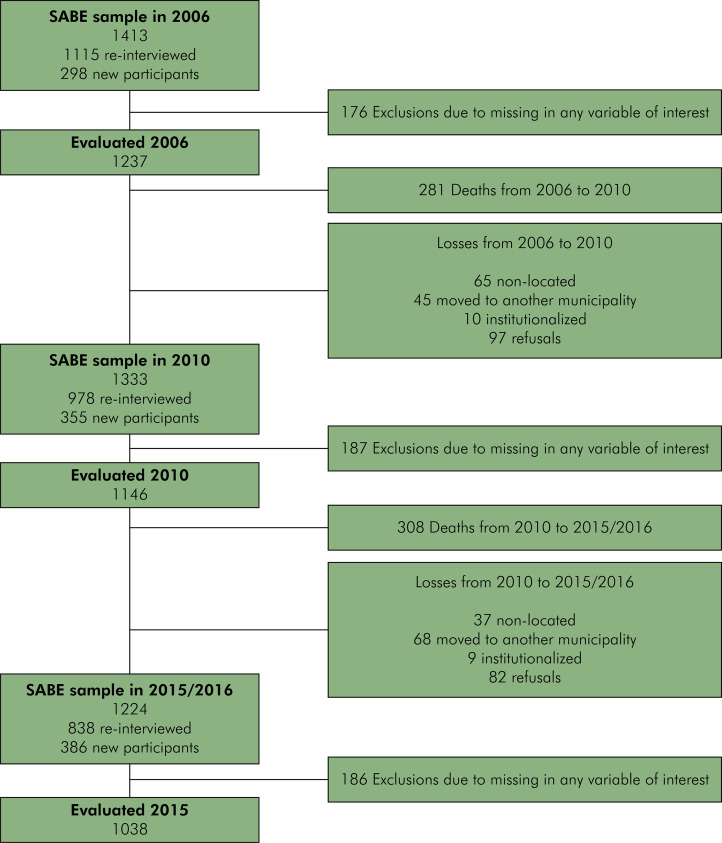
Sample size according to study waves.

### Data collection

All aspects of data collection for the SABE study were conducted in the participants’ homes, including the administration of questionnaires, clinical and physical examinations, and biological material collection. The questionnaires were administered by trained interviewers, and the dental clinical examinations were performed by dentists who underwent both theoretical and clinical training specifically for the study in all waves.

### Ethical considerations

The SABE study was approved by the Research Ethics Committee of the School of Public Health, University of São Paulo (no. 67/99 for the 2000 wave; 83/06 for the 2006 wave; 23/10 for the 2010 wave); and 3,600,782 for the 2015 wave), ensuring compliance with ethical standards and protocols. Prior to the interviews in each wave, written informed consent was obtained from all participants.

### Dependent variable

Perceived mobility limitation was the outcome of the study, and it was defined according to reports of difficulty in performing seven activities previously described in the literature:^15-17^ a) pulling or pushing large objects; b) lifting or carrying weights greater than 5 kg; c) climbing several flights of stairs; d) climbing a single flight of stairs; e) stooping, kneeling, or crouching; f) walking several blocks; and g) walking one block. Individuals who reported being unable to perform the activities were considered to have mobility limitations. The limitations were summed to form a score ranging from 0 to 7, with higher scores representing a higher number of mobility limitations. This variable was assessed in every wave.

### Independent variables of interest

The independent variables of interest were number of teeth (0, 1–9, 10–19, 20 or more), use of dental prostheses (no, yes), impact of oral health on functionality (no, yes), and presence of periodontal pocket ≥ 4 mm (no, yes). Dentition status was evaluated by clinical oral health examination performed by trained dentists using the World Health Organization protocol for oral health surveys.^18^ Oral health measures were evaluated in all waves of the study.

The assessment of the self-perceived impact of oral health on functionality was conducted through the functional dimension of the Geriatric Oral Health Assessment Index.^19^ This tool consists of 12 items aimed at evaluating issues related to the impact of oral health across three domains: physical functionality, psychosocial effects, and pain or discomfort. Within the physical functionality domain, four questions focus on difficulties in eating, speaking, swallowing, and pronouncing words due to dental or denture issues. These questions utilized a 5-point Likert scale, asking participants about the frequency of these problems over the past year, ranging from “always” to “never.” Those indicating they “always” or “often” encountered such issues were classified as having an impact on oral health and functionality.

### Covariates

The study accounted for the following covariates: sociodemographic factors (age at baseline [age when the participant began participating in the study], sex, and years of education); lifestyle behaviors (smoking status [none, current, former], engagement in physical activities [no, yes], and alcohol consumption [none, yes/but not at risk of alcoholism, yes/at risk of alcoholism]); and overall health conditions (presence or absence of hypertension, diabetes, cardiovascular diseases, stroke, cognitive performance, and body mass index calculated as weight in kilograms divided by height in squared meters). Covariates were also assessed in all waves, except for age and sex.

Physical activity was determined based on whether participants engaged in at least 150 minutes of moderate activity or 75 minutes of vigorous activity weekly^20^ and was evaluated using the International Physical Activity Questionnaire.^21^ Cognitive performance was assessed using the Mini-Mental State Examination, which was adapted and validated for the SABE study.^22^ Alcohol consumption was evaluated with the Short Michigan Alcoholism Screening Test, categorizing individuals as at-risk or not-at-risk for alcoholism.^23^ Self-reported chronic conditions were verified through previous diagnoses by healthcare professionals.

### Statistical analysis

The data analyses were performed using R 4.2.3 software. The effect of oral health conditions on mobility limitation was assessed using the generalized linear mixed models. In these models, the differences between groups can be modeled as a random effect, and they are useful in the analysis of longitudinal data. A Poisson distribution was applied, given that the dependent variable is a count variable. The link function for this distribution was the log. No overdispersion was observed in the data, indicating that the Poisson distribution fitted the data well. The data were analyzed using the “glmer” command from the “lme4” R package. Estimates were presented as an incidence rate ratio (IRR) and their 95% confidence intervals (95% CI). All the variables in the model were treated as time-varying, except for age and sex. An interaction term between the dentition status variables and time was included in the final model. The analysis of the presence of periodontal pockets was restricted to individuals with natural teeth. Because blood sample data were only available from the third (2010) and fourth (2015) waves, supplementary analyses were performed to investigate the influence of C-reactive protein (mg/L) and albumin (g/dL) levels on the relationship between oral health status and mobility limitation.

## Results

This study evaluated 1,903 individuals, corresponding to 3,293 observations. For the total sample analyses, 365 participants had data from three waves, 660 from two waves, and 878 from one wave. The descriptive analysis within each wave shows that 12.9%, 17.4% and 20.9% of the participants had 20 or more teeth in 2006, 2010, and 2015, respectively. Most of them wore dental prostheses in each wave and had mobility limitations (Table 1).

**Table 1:** Distribution of oral health measures and mobility limitation across study waves. (São Paulo, Brazil, 2006, 2010 and 2015.

Variable	Wave 2 (2006)	Wave 3 (2010)	Wave 4 (2015)
	% (95%CI)	% (95%CI)	%(95%CI)
	n = 1,237	n = 1,146	n = 1,038
Number of teeth			
0	51.6 (48.8–55.6)	42.8 (39.8–45.9)	35.4 (32.2–38.7)
1–9	21.6 (18.7–24.5)	22.5 (19.5–25.6)	23.8 (20.6–27.1)
10–19	13.9 (11.1–16.8)	17.3 (14.2–20.4)	19.9 (16.8–23.3)
≥ 20	12.9 (10.0–15.8)	17.4 (14.3–20.4)	20.9 (17.7–24.2)
Use of dental prosthesis			
No	17.9 (15.8–20.0)	21.1 (18.8–24.5)	21.8 (19.3–24.3)
Yes	82.1 (79.9–84.2)	79.9 (76.7– 81.4)	78.2 (75.7–80.7)
Periodontal pocket^*^			
No	82.8 (79.9–85.8)	63.5(59.8–67.4)	53.9 (50.1–57.9)
Yes	17.2 (14.4–20.3)	36.5 (32.8–40.4)	46.1 (42.2–50.1)
Impact of oral health on functionality			
No	65.2 (62.5–67.9)	67.3 (64.6–70.2)	76.9 (74.4–79.5)
Yes	34.8 (32.2–37.6)	32.7 (30.0–35.6)	23.1 (20.6–25.8)
Mobility limitation			
No (score = 0)	21.7 (19.4–23.9)	25.3 (22.8–27.9)	28.1 (25.4–30.9)
Yes (score ≥ 1)	78.3 (76.1–80.7)	74.7 (72.2–7.2)	71.9 (69.2–74.7)

^*^Estimation for dentate individuals.

Table 2 shows the longitudinal analysis of the associations between the dentition status variables and mobility limitation, considering the total sample. In the unadjusted models, both the number of teeth and the self-reported impact of oral health on functionality were associated with mobility limitation. In the fully adjusted model (model 3), only individuals with 20 or more teeth [IRR: 0.88, 95%CI 0.78–0.99] had a lower risk of mobility limitations than those who were edentulous. Those who reported an impact of oral health on functionality had a higher risk of limitation than older adults who did not report such an impact [IRR: 1.25; 95%CI 1.18–1.32]. There were no significant interactions between the number of teeth and time or the impact of oral health on functionality and time.

**Table 2:** Longitudinal association between dentition status and mobility limitation (outcome) for the total sample. São Paulo, Brazil, 2006, 2010, and 2015.

Fixed effects	Model 1		Model 2		Model 3	
	IRR (95%CI)	p-value	IRR (95%CI)	p-value	IRR (95%CI)	p-value
Number of teeth (ref = 0)						
1–9	0.82 (0.75–0.90)	< 0.001	1.05 (0.97–1.14)		1.04 (0.96–1.13)	
10–19	0.65 (0.59–0.72)	< 0.001	0.93 (0.84–1.03)		0.95 (0.86–1.05)	
≥ 20	0.48 (0.43–0.54)	< 0.001	0.83 (0.73–0.94)	p < 0.01	0.88 (0.78–0.99)	p < 0.05
Use of dental prosthesis (ref = no)						
Yes	1.07 (0.98–1.16)	< 0.001	0.92 (0.84–1.00)		0.95 (0.87–1.04)	
Impact of oral health on functionality (ref = no)						
Yes	1.35 (1.28–1.43)	< 0.001			1.25 (1.18–1.32)	< 0.001
Time (ref = 2006)						
2010	1.01 (0.96–1.07)		0.96 (0.91–1.01)		0.95 (0.89–1.00)	
2015	1.15 (1.08–1.21)	< 0.001	1.07 (1.00–1.14)		1.08 (1.01–1.15)	p < 0.05
Random effects						
σ^2^			6.19		6.19	
ID (intercept) variance			0.29		0.27	

IRR: Incidence rate ratio; ref: reference category. Model 1: unadjusted + time; Model 2: Model adjusted for number of teeth, use of dental prostheses, age at baseline, and time-varying covariates (sex, schooling, hypertension, diabetes, cardiovascular, disease, stroke, smoke, alcohol, physical activity, cognitive performance, and BMI); Model 3: Model 2 + impact of oral health on functionality.

The analyses for dentate individuals included 1,883 observations from 1,149 individuals. Of these, 187 had data from three waves, 360 from two waves, and 602 from one wave. There was no association between the presence of periodontal pockets and mobility limitation (Table 3). In the adjusted model (model 3), a negative risk was found between the number of teeth and the mobility limitation score, indicating that older adults with 20 or more teeth had a lower risk of mobility limitations. The impact of oral health on functionality increased the risk of mobility limitations (model 3).

**Table 3:** Longitudinal association between dentition status and mobility limitation score (outcome) among dentate individuals. São Paulo, Brazil, 2006, 2010, and 2015.

Variables	Model 1		Model 2		Model 3	
	IRR (95%CI)	p-value	IRR (95%CI)	p-value	IRR (95%CI)	p-value
Number of teeth (ref.= 1-9)						
10–19	0.81 (0.72–0.91)	< 0.01	0.93 (0.83–1.04)		0.94 (0.84–1.05)	
≥ 20	0.59 (0.52–0.68)	< 0.001	0.86 (0.75–0.99)	< 0.05	0.87 (0.75–1.00)	< 0.05
Use of dental prosthesis (ref = no)						
Yes	1.07 (0.96–1.20)		0.95 (0.85–1.06)		0.94 (0.84–1.05)	
Impact of oral health on functionality (ref = no)						
Yes	1.35 (1.24–1.48	< 0.001	1.28 (1.17–1.40)	< 0.001	1.28 (1.18–1.40)	< 0.001
Periodontal pocket (ref = no)						
Yes	0.88 (0.81–0.97)				0.95 (0.87–1.04)	
Time (ref = 2006)						
2010	0.98 (0.90–1.05)		0.88 (0.81–0.96)	< 0.01	0.89 (0.82–0.97)	< 0.01
2015	1.15 (1.05–1.25)	< 0.01	1.04 (0.94–1.15)		1.05 (0.95–1.17)	
Random effects						
σ^2^			6.42		6.42	
ID (intercept) variance			0.36		0.36	

ref: reference category; IRR: Incidence rate ratio. Model 1 = unadjusted + time; Model 2 = Model adjusted for number of teeth, use of dental prostheses, impact of oral health on functionality, age at baseline, and time-varying covariates (sex, schooling, hypertension, diabetes, cardiovascular, disease, stroke, smoke, alcohol, physical activity, cognitive performance, and BMI; Model 3 = Model 2 + periodontal pocket.

In additional analyses (Tables 4 and 5), only the impact of oral health on functionality was associated with mobility limitations. The adjustment for albumin and C reactive protein did not affect the magnitude of the previous association.

**Table 4:** Longitudinal association between oral health and mobility limitation score for the total sample (2010-2015).

Variable	Model 1		Model 2	
	IRR (95%CI)	p-value	IRR (95%CI)	p-value
Number of teeth (ref = 0)				
1–9	1.02 (0.93–1.13)		1.03 (0.93–1.14)	
10–19	0.93 (0.83–1.05)		0.93 (0.83–1.05)	
≥ 20	0.86 (0.74–0.99)		0.87 (0.75–1.01)	
Use of dental prosthesis (ref = no)				
Yes	0.93 (0.83–1.04)		0.92 (0.82–1.03)	
Impact of oral health on functionality (ref = no)				
Yes	1.32 (1.22–1.42)	< 0.001	1.33 (1.23–1.44)	< 0.001
C-reactive protein			1.01 (1.00–1.01)	< 0.05
Albumin			0.85 (0.75–0.97)	< 0.05
Time (ref = 2010)				
2015	1.17 (1.10–1.24)	< 0.001	1.16 (1.09–1.24)	< 0.001
Random effects				
σ^2^	6.24		6.24	
ID (intercept) variance	0.29		0.28	

1,001 observations from 730 individuals. Of these, 271 had data from two waves and 459 had data from one wave. São Paulo, Brazil, 2006, 2010, and 2015.

ref: reference category; IRR: Incidence rate ratio. Model 1 = adjusted for age, sex, schooling, hypertension, diabetes, cardiovascular disease, stroke, smoke, alcohol, physical activity, cognitive performance, and BMI.; Model 2 = model 1 + C-reactive protein and albumin.

**Table 5:** Longitudinal association between oral health and mobility limitation score among dentate individuals (2010-2015).††

Variable	Model 1		Model 2	
	IRR (95%CI)	p-value	IRR (95%CI)	p-value
Number of teeth (ref = 1–9)				
10–19	0.95 (0.83–1.09)		0.94 (0.82–1.09)	
≥ 20	0.89 (0.75–1.05)		0.90 (0.76–1.06)	
Use of dental prosthesis (ref = no)				
Yes	0.97 (0.85–1.11)		0.97 (0.84–1.11)	
Impact of oral health on functionality (ref = no)				
Yes	1.41 (1.26–1.57)	< 0.001	1.44 (1.28–1.61)	< 0.001
Periodontal pocket (ref = no)				
Yes	0.93 (0.84–1.04)		0.94 (0.84–1.04)	
C-reactive protein			1.00 (1.00–1.01)	
Albumin			0.84 (0.70–1.02)	
Time (ref = 2010)				
2015	1.23 (1.12–1.34)	< 0.001	1.22 (1.11–1.34)	< 0.001
Random effects				
σ^2^	6.44		6.44	
ID (intercept) variance	0.36		0.35	

1,001 observations from 730 individuals. Of these, 271 had data from two waves and 459 had data from one wave. São Paulo, Brazil, 2006, 2010, and 2015.

IRR: incidence rate ratio; ref: reference category. Model 1 = adjusted for age, sex, schooling, hypertension, diabetes, cardiovascular disease, stroke, smoke, alcohol, physical activity, cognitive performance, and BMI; Model 2 = Model 1 + C-reactive protein and albumin.

## Discussion

This study evaluated the effect of dentition status, measured at three time points over 10 years, on the perceived mobility limitation among community-dwelling Brazilian older adults. Our findings show that older adults with 20 or more teeth had a lower risk of mobility limitation than edentulous individuals. Additionally, the perceived impact of oral health on functionality increased the risk of mobility limitations. Similar findings were observed among dentate individuals. In dentate individuals, periodontal disease was not associated with the outcome. In all models, the findings suggest that the risk between oral health measures and mobility limitation is constant over time.

Our findings corroborate previous research involving individuals aged 70 years and older.^12,13^ However, the associations differed heterogeneously across the categories of teeth. In line with the present study, the Health, Aging, and Body Composition (HABC) study^12^ found that complete tooth loss (0 versus ≥21 natural teeth) increased the odds of developing mobility limitations. In the British Regional Heart Study (BRHS)^12^ and in the study from the Center of Preventive Medicine in Glostrup, Denmark,^13^ complete tooth loss was not associated with partial mobility. In BHRS,^12^ partial tooth loss (8–14 or 15–20 natural teeth versus ≥ 21 teeth) was associated with a higher incidence of mobility limitations. In the Danish study,^13^ only individuals with 1–9 teeth had a higher risk of developing mobility limitation than older adults with 20 or more teeth.

The present study also adds to the evidence by showing the independent negative effect of the perceived impact of oral health conditions on functionality. Self-reported health measures complement clinical measures and reflect individuals’ perceptions and evaluations of their health.^24^ In line with these findings, the English Longitudinal Study of Aging demonstrated that tooth loss affects the incidence of limitation in activities of daily living, mainly through self-reported eating difficulty.^25^ Among adults aged 50 years and older, locomotive function was only associated with chewing function in analyses that considered the number of teeth and chewing function simultaneously, indicating that chewing function is more closely related to the outcome.^26^ Additionally, individuals with impaired chewing ability have been found to have lower muscle strength.^27^ Another study found that prosthetic rehabilitation alone was not associated with improved postural balance, which was observed only among patients who also showed improved masticatory capacity.^28^

In this context, our findings and previous evidence linking oral health to mobility limitations might be explained by the impact of chewing difficulties — resulting from tooth loss —on food choices and overall nutrition.^7,29^ Poor diet and compromised nutritional status have been identified as risk factors for sarcopenia,^30-32^ which, in turn, affects physical function and is an important component of intrinsic capacity.^3^

Finally, periodontal disease was not associated with mobility limitations in the present study. Inflammation caused by periodontal disease has been suggested as a mediator in this association,^8^ as it can lead to loss of muscle mass and strength, both of which are associated with a decline in mobility.^33,34^ This pathway has been underexplored and the findings have been inconsistent. Among British adults aged 70 years and older, participants with 20% or more sites showing loss of attachment >5.5 mm had a higher risk of mobility limitation, defined as difficulty going up or down stairs or walking 400 yards. In contrast, among American adults aged 70 years and older, the use of 20% or more of sites with loss of attachment >3 mm did not reveal any association. Mobility difficulty was defined as difficulty walking one quarter of a mile or climbing one flight of stairs or both. Neither study found an association between pocket depth and the incidence of disability.^12^

One of the strengths of this study is the use of data from a diverse population in Brazil’s largest city. Additionally, considering that this study was designed to evaluate the aging process, the analyses could be adjusted for a comprehensive number of confounders evaluated in each wave. Time-varying covariates allowed us to adjust for changes in the status of these variables and oral health during the follow-up periods. Another strength is the use of clinically assessed oral health measures. The higher prevalence of severe tooth loss, however, might have affected the evaluation of the effect of periodontal pockets. Moreover, edentulism at younger ages may be associated with adaptations in the ability to eat, which could attenuate the association. Although we treated the variables as time-varying to account for changes in status during the study period, the long intervals between follow-ups should also be considered when interpreting the findings.

## Conclusions

The findings of our study underscore the importance of maintaining functional dentition to reduce mobility limitations in older adults. They also highlight the importance of evaluating the perceived impact of oral health in addition to the number of teeth. Given that severe tooth loss is still highly prevalent among older cohorts, appropriate oral health rehabilitation should be further investigated to mitigate health impacts in this age group. The findings also underscore that public policies and healthcare systems should consider integrating dental care into broader health strategies for the older population.

## Data Availability

The contents underlying the research text are contained in the manuscript.
